# Understanding Nursing Home Responses to Minnesota’s New Value Based Reimbursement System

**DOI:** 10.1093/geroni/igaa057.285

**Published:** 2020-12-16

**Authors:** Zachary Hass, Kathleen Abrahamson, Dongjuan Xu, Valerie Cooke

**Affiliations:** 1 Purdue University, West Lafayette, Indiana, United States; 2 Minnesota Department of Human Services, St. Paul, Minnesota, United States

## Abstract

Even though Value Based Reimbursement (VBR) systems for nursing homes (NH) continue to expand, we have little understanding of how NH respond to VBR. In 2016, Minnesota passed VBR legislation for NHs that increased care-related funding and tied increases to a facility’s composite quality score. While care-related expenditures increased with VBR, the incentive for quality did not work as intended. We investigated the differential responses of facilities in their care-related expenditures and quality scores. Data were derived from cost reports and quality measures for the years 2013-2017 from 300 free-standing Minnesota NHs. Latent Class Growth Analysis was used to cluster facilities by their joint care-related cost and quality score trajectories over the period. Three interpretable trajectory clusters emerged: medium-to-high cost and medium-to-high quality (n=172), low cost and medium-to-high quality (n=54), and low cost and low quality (n=74), all during the pre-VBR period. In all three clusters cost rose significantly with VBR, but only in the low cost and low quality cluster did quality also rise significantly. The quality improving cluster had the highest percentage of government-owned and rural facilities as well as the largest annual increase in care related spending. The medium-to-high cost and medium-to-high quality cluster had the highest concentration of urban facilities (Twin City Metro Area) and were the most likely to be non-profit and chain owned. Although the new VBR system appeared effective in achieving its goals for a subset of facilities with lowest cost and quality, the majority of facilities increased care-related costs without improved quality.

